# Nutritional Risk and Health-Related Quality of Life in Older Adults Aging With HIV

**DOI:** 10.1093/geroni/igab046.3418

**Published:** 2021-12-17

**Authors:** Nicole Viviano, Ann Gruber-Baldini, Sarah Schmalzle, Kristen Stafford, Sarah Chard, Kareshma Mohanty, Elizabeth Parker, Uzoamaka Eke

**Affiliations:** 1 University of Maryland, Baltimore, Gaithersburg, Maryland, United States; 2 University of Maryland School of Meidcine, Baltimore, Maryland, United States; 3 University of Maryland School of Medicine, Baltimore, Maryland, United States; 4 UMBC, Baltimore, Maryland, United States

## Abstract

Due to antiretroviral treatment success, individuals with HIV are living longer. People aging with HIV (PAWH, 50+) may be more likely to experience nutritional risk compared to their HIV-negative counterparts due to biopsychosocial factors. The DETERMINE checklist measure accounts for social and economic factors as well as aspects of the aging process that are not typically considered when examining nutritional risk and are important for PAWH. The current study examined nutritional risk and health-related quality of life (HRQoL) in PAWH using the DETERMINE checklist and PROMIS t-scores (mental and physical HRQoL) through secondary analyses of 158 participants in the Strengthening Therapeutic Resources in Older patients agiNG with HIV (STRONG) study. DETERMINE nutritional risk scores (0-21) were separated into 4 groups (low-risk [0-2, n=13], moderate-risk [3-5, n=28], high-risk [6-12, n=78], very high-risk [13-21, n=39]). The sample was 55% male, 94% Black/African American and had a mean age=59 (SD=5.5). Most of the sample (74%) were at high or very high nutritional risk and low HRQoL t-score: physical M=43.7 (SD=9.5), and mental M=45.7 (SD=10.1). Mental and physical HRQoL were significantly (p<.001) associated with nutritional risk group as tested through linear regressions. Means were as follows: physical HRQoL low-risk M=53.4 (SD=10.6), moderate-risk M=47.4 (SD=8.9), high-risk M=43.5 (SD=8.1), very high-risk M=38.4 (SD=8.9); mental HRQoL low-risk M=54.0 (SD=8.9), moderate-risk M=49.1(SD=7.9), high-risk M=46.1(SD=9.5), and very high-risk M=39.5 (SD=9.7). These associations remained significant after controlling for age and sex. Higher nutritional risk as measured by the DETERMINE checklist in PAWH was associated with poorer physical and mental HRQoL.

